# Association between clinical characteristics within 6 h of ICU admission and 30-day mortality risk in immunocompromised sepsis patients: development and validation of a machine learning model based on the MIMIC-IV database

**DOI:** 10.3389/fdgth.2026.1881138

**Published:** 2026-07-13

**Authors:** Zhipeng Cheng, Xiuqing Ma, Weiying Duan, Zeyu Mou, Zhixin Liang

**Affiliations:** Department of Respiratory and Critical Care Medicine, First Medical Center, Chinese PLA General Hospital, Beijing, China

**Keywords:** eICU-CRD, immunosuppression, MIMIC-IV, mortality risk, prediction model, sepsis, support vector machine

## Abstract

**Objective:**

To develop and validate a machine learning model for predicting 30-day mortality in immunocompromised sepsis patients using clinical data within 6 h of ICU admission.

**Methods:**

This retrospective cohort study utilized data from the MIMIC-IV and eICU databases. Adult immunosuppressed patients meeting Sepsis-3 criteria were enrolled, and clinical indicators within 6 h of ICU admission were extracted. Variable selection was performed using multiple testing correction and recursive elimination algorithms, ultimately incorporating 10 feature variables. Predictive models were constructed using seven machine learning algorithms: Logistic Regression, Decision Tree, Random Forest, XGBoost, LightGBM, Support Vector Machine (SVM), and Artificial Neural Network (ANN). Model discriminative capabilities were assessed using Area Under the Receiver Operating Characteristic Curve (AUC), calibration curves, and Decision Curve Analysis (DCA). The best-performing model underwent external validation using eICU database data and SHAP interpretability analysis.

**Results:**

A total of 2,494 immunosuppressed sepsis patients were included, with a 30-day mortality rate of 33.4%. The final prediction model included 10 feature variables: Weight, APS-III score, Urine output, Prothrombin Time (PT), Blood Urea Nitrogen (BUN), SOFA score, Red Blood Cell count (RBC), Platelet count (PLT), Age, and Mean Corpuscular Hemoglobin Concentration (MCHC). Among all tested machine learning algorithms, the Support Vector Machine (SVM) model demonstrated the best predictive performance, with an AUC of 0.794 (95% CI: 0.761–0.826) in the validation set. Calibration curves and DCA showed good consistency between predicted probabilities and actual risk. External validation on the eICU dataset (*n* = 1,280) yielded an AUC of 0.847, indicating stable predictive performance and strong generalizability.

**Conclusion:**

This study successfully developed an SVM-based prediction model that effectively predicts 30-day mortality risk in immunosuppressed sepsis patients using only 10 easily obtainable clinical indicators available within 6 h of admission. This model shows promise as a practical tool for early identification of high-risk immunosuppressed sepsis patients and assisting in personalized treatment decisions in clinical settings.

## Background

Sepsis is a life-threatening organ dysfunction caused by a dysregulated host response to infection and is one of the leading causes of death in intensive care unit (ICU) patients worldwide (1). Within the broader sepsis population, there exists a particularly vulnerable subgroup—immunosuppressed patients. The pathophysiological processes in these patients differ significantly from those in non-immunosuppressed patients. On one hand, pathogens may proliferate more uncontrollably; on the other hand, after an initial hyperinflammatory phase, the body often rapidly transitions into a prolonged immunosuppressive state, characterized by increased lymphocyte apoptosis and immune paralysis, leading to heightened susceptibility to secondary infections and ultimately a significantly increased risk of death (2, 3). Studies have shown that immunosuppression is an independent risk factor for mortality in sepsis patients; the clinical course in these patients is more insidious, treatment response is poorer, and mortality is higher (4). However, prognostic scoring systems widely used in the general sepsis population (such as SOFA, APACHE II, etc.) may have limited predictive efficacy in this special immunosuppressed group (5). Therefore, there is an urgent need to develop specific risk prediction tools tailored for immunosuppressed sepsis patients.

Sepsis treatment emphasizes the “golden hour,” meaning effective intervention early in the course can maximize patient outcomes. Risk stratification based on early clinical data (e.g., within 6 h of admission) can help clinicians promptly identify patients who appear stable but are actually at high risk, thereby enabling rational allocation of medical resources and implementation of intensive monitoring and personalized treatment. In recent years, machine learning technology has shown great potential in the field of medical prediction. Compared to traditional logistic regression models, machine learning algorithms (such as SVM, Random Forest, etc.) can better capture complex nonlinear relationships among multiple predictor variables, potentially offering more accurate predictive performance (6). Among them, Support Vector Machine (SVM), as a powerful classification algorithm, finds the optimal hyperplane that maximizes the margin between two classes of samples, demonstrating good generalization ability and high accuracy when handling high-dimensional data, and has been successfully applied in prediction models for various diseases (7).

Therefore, this study aims to utilize large public databases (MIMIC-IV and eICU) to specifically develop and validate a machine learning framework for predicting 30-day mortality risk in immunosuppressed sepsis patients, based strictly on clinical data available within the first 6 h of ICU admission. By systematically comparing multiple machine learning algorithms, we intend to provide clinicians with a reliable and easily obtainable early risk-stratification tool for this highly vulnerable population.

## Materials and methods

### Data sources and population selection

This was a retrospective cohort study based on data from the Medical Information Mart for Intensive Care IV (MIMIC-IV version 3.1) and the eICU Collaborative Research Database (eICU-CRD version 2.0). MIMIC-IV (v3.1) is a large, single-center public database containing data from all ICU and emergency department patients at Beth Israel Deaconess Medical Center in Boston, Massachusetts, USA, between 2008 and 2019 (8). The eICU-CRD (v2.0) is a freely available multi-center critical care research database containing clinical data from over 200,000 patients admitted to 335 ICUs across 208 hospitals in the USA between 2014 and 2015 (9). This study analyzed publicly available, de-identified data from the MIMIC-IV and eICU Collaborative Research Databases. Our research team has obtained the necessary institutional authorization to access and utilize these databases. As the data are de-identified and publicly accessible, institutional review board (IRB) approval and informed consent were waived and a formal study protocol was not prospectively registered. All procedures were conducted in accordance with the Declaration of Helsinki. To ensure compliance with ethical standards, the primary investigator (Cheng Zhipeng) completed the Collaborative Institutional Training Initiative (CITI) training (Record ID: 73074624).

Inclusion criteria: (1) age >18 years; (2) diagnosis of sepsis on the day of ICU admission; (3) meeting the definition of immunosuppression (see below).

Exclusion criteria: (1) ICU length of stay ≤24 h; (2) Missing data ≥20% of variables.

### Definitions

Sepsis was defined according to the Sepsis-3 criteria jointly released by the American Thoracic Society (ATS) and the Society of Critical Care Medicine (SCCM) in 2016, i.e., confirmed or suspected infection and a Sequential Organ Failure Assessment (SOFA) score increase of >2 points (1).

Immunosuppression was defined as the presence of at least one of the following conditions, aligned with established clinical research benchmarks: 1. Neutropenia: Absolute neutrophil count (ANC) ≤0.5 × 10^9^/L at the time of admission or within 60 days prior. 2. Chronic Glucocorticoid Therapy: Receipt of a prednisone-equivalent dose of ≥20 mg/day for ≥3 weeks at the time of disease onset. 3. Immunosuppressive Therapy: Receipt of chemotherapy, biological agents, or maintenance immunosuppressants for transplantation within 90 days before sepsis onset. 4. Transplantation: History of allogeneic hematopoietic stem cell transplantation (HSCT) or solid organ transplantation requiring ongoing immunosuppression. 5. Other Conditions: Presence of graft-vs.-host disease or severe congenital/acquired immunodeficiency syndromes (e.g., HIV/AIDS with low CD4+ count) (10, 11).

### Data extraction

Researchers extracted data from the first 6 h of ICU admission from both databases, including demographic data, laboratory indicators, comorbidities, and treatment measures, as potential predictors. Demographic characteristics included age, sex, height, and weight. Comorbidities were identified and extracted based on International Classification of Diseases, Ninth Revision (ICD-9) and Tenth Revision (ICD-10) diagnosis codes, including hypertension, diabetes, chronic liver disease, chronic kidney disease, chronic heart disease, chronic lung disease, cerebrovascular disease, cancer, metastatic solid tumor, rheumatoid disease. The maximum value of laboratory indicators should be extracted within 6 h of admission, which included White Blood Cell count (WBC), Red Blood Cell count (RBC), Platelet count (PLT), Hematocrit (HCT), Mean Corpuscular Hemoglobin (MCH), Mean Corpuscular Volume (MCV), Mean Corpuscular Hemoglobin Concentration (MCHC), Serum Creatinine (Cr), Blood Urea Nitrogen (BUN), Prothrombin Time (PT), Partial Thromboplastin Time (PTT), International Normalized Ratio (INR), Alanine Aminotransferase (ALT), Aspartate Aminotransferase (AST), and 6-h Urine output (Urine). Treatment measures included use of Vasoactive Agents, Invasive Ventilation, Continuous Renal Replacement Therapy (CRRT), and Renal Replacement Therapy (RRT). The Acute Physiology Score III (APS-III), Simplified Acute Physiology Score II (SAPS-II), Sequential Organ Failure Assessment (SOFA) scores, Glasgow Coma Scale (GCS) and Charlson Comorbidity Index (CCI) were calculated using variables extracted within specific windows post-ICU admission. The outcome variable was 30-day in-hospital mortality.

### Data processing and model construction

**Figure 1:**
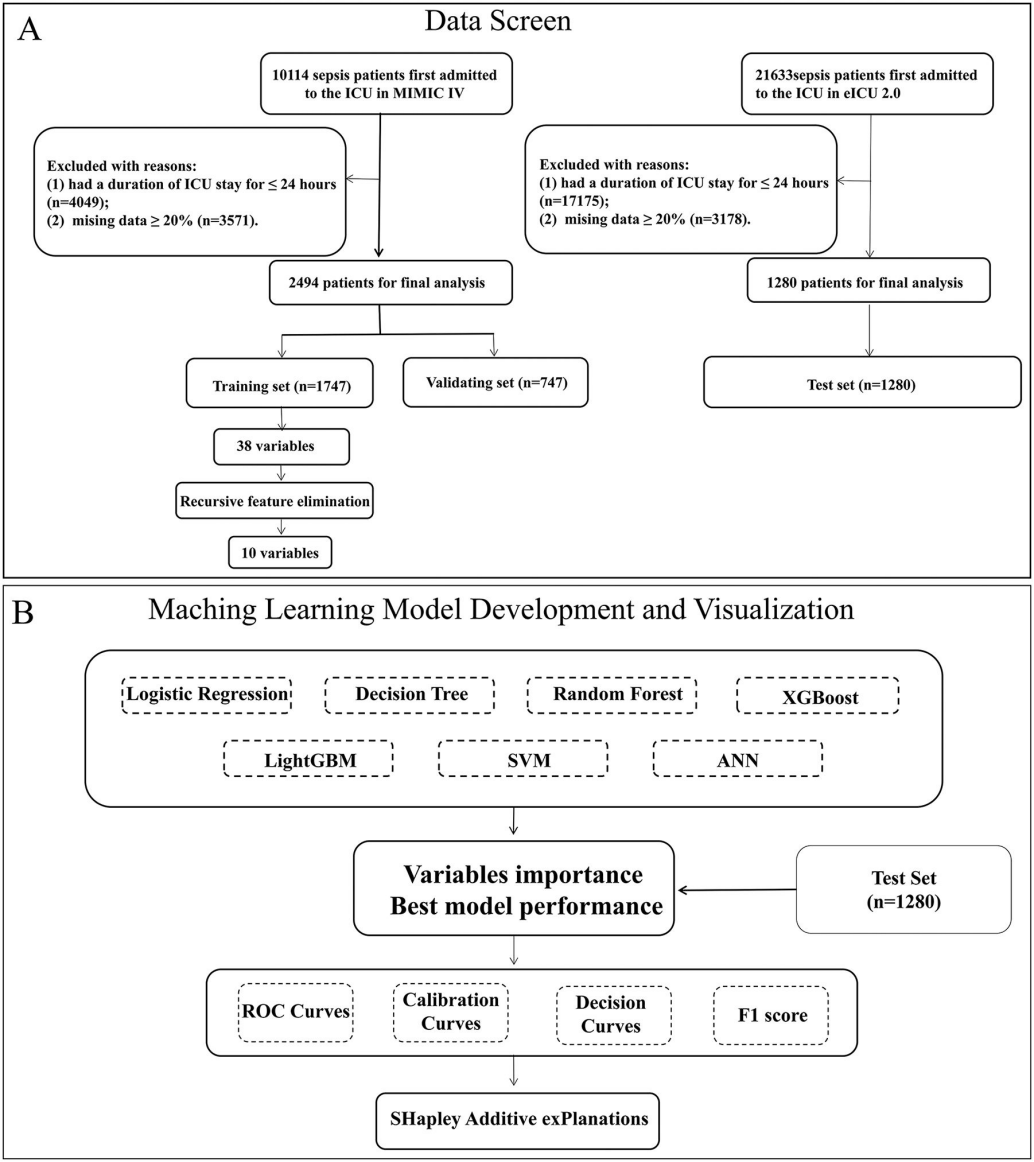
Flow diagram detailing the retrospective patient screening, selection criteria, and cohort establishment based on Sepsis-3 guidelines across the MIMIC-IV and eICU databases. **(A)** Flowchart of patient selection; **(B)** Diagram of machine learning workflow.

**Figure 2:**
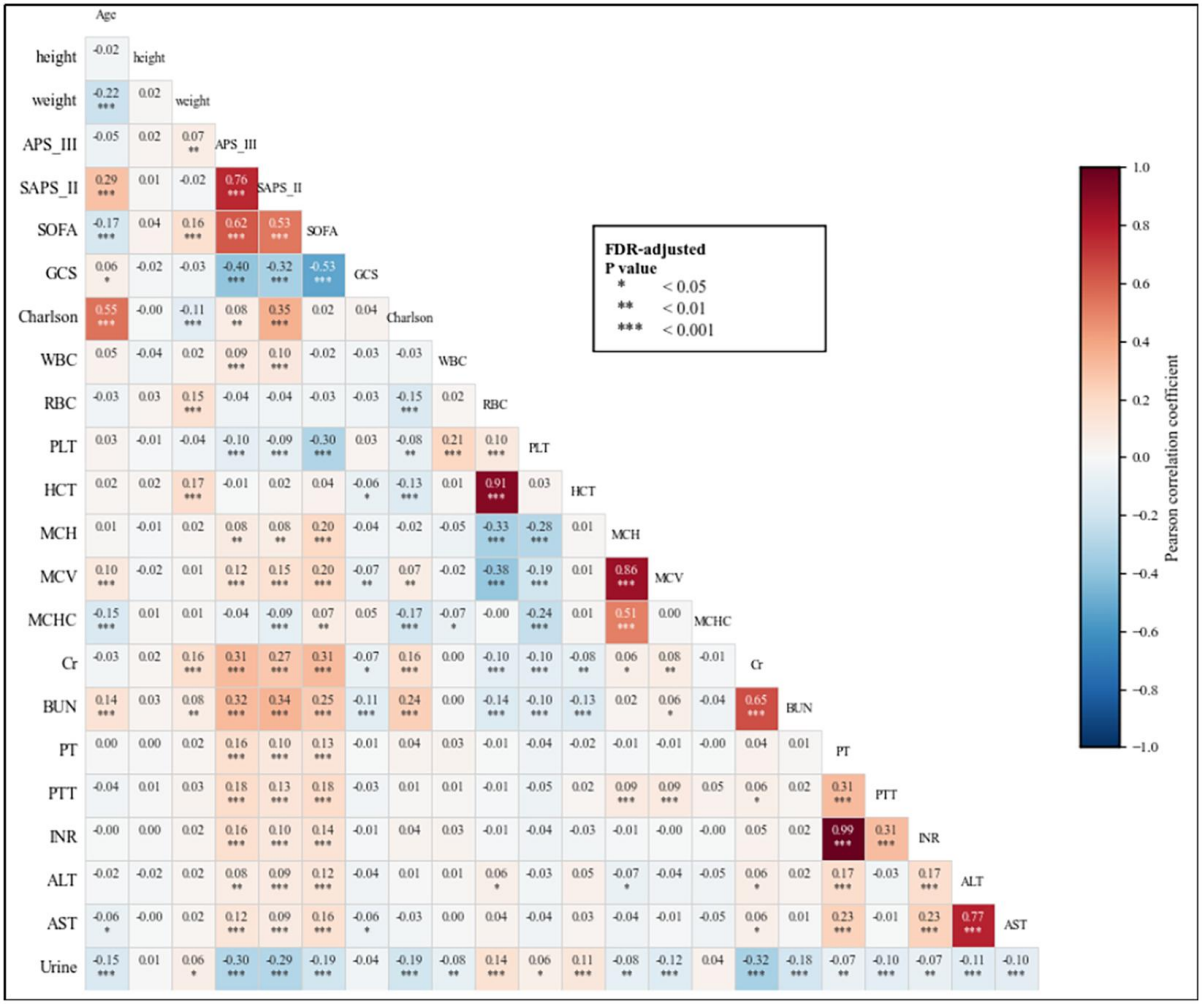
Results of multiple testing correction among variables.

**Figure 3:**
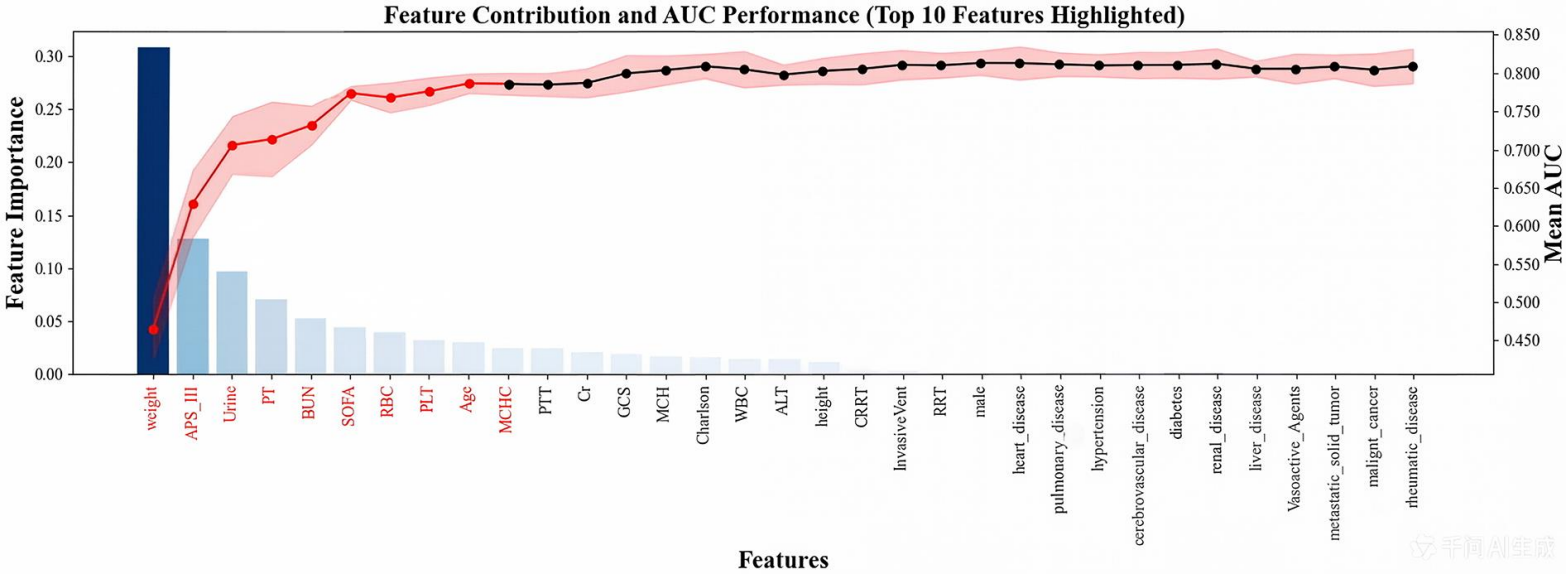
Results of variable selection using recursive feature elimination.

The data processing and model construction workflow is shown in Figure 1. This study included 2,494 immunosuppressed sepsis patients from the MIMIC-IV database and 1,280 from the eICU database. The sample size was determined by data availability. The derivation cohort included 833 events and 10 predictors, yielding an events-per-variable (EPV) ratio of 83.3. This safely exceeds the recommended threshold of 10–20, ensuring adequate statistical power and minimizing overfitting. To reduce bias caused by missing data, the VIM package in R (version 4.5.1) was used to identify the distribution of missing values (Supplementary Figure S2), and features with more than 20% missing values were excluded. For missing values in the remaining variables, we used the mice package in R for multiple imputation. This method considers inter-variable relationships to assign multiple plausible values for missing data, generating 5 imputed datasets. One complete dataset was randomly selected for subsequent analysis. The 2,494 cases from MIMIC-IV were randomly split into a training set and a validation set in a 7:3 ratio (12). The 1,280 cases from the eICU database served as the external validation set. This study initially included 1 outcome variable and 38 clinical indicator variables. Through multiple testing correction, Pearson correlation coefficients (*r*) and False Discovery Rate adjusted *P*-values (FDR-P) were calculated for all variable pairs (Figure 2). One variable from any pair with |*r*| > 0.7 and FDR-P < 0.05 was excluded. The remaining variables underwent feature selection using Recursive Feature Elimination (RFE) to determine the optimal feature subset (Figure 3). The final selected optimal feature variables were: Weight, APS-III score, Urine output, PT, BUN, SOFA score, RBC, PLT, Age, and MCHC. Multiple testing correction and RFE were performed using Python 3.10 software.

This study employed seven machine learning algorithms to predict 30-day mortality risk in immunosuppressed sepsis patients: Logistic Regression, Decision Tree, Random Forest, XGBoost, LightGBM, SVM, and ANN. Optimal hyperparameters for each model were determined via grid search (13). In the training set, k-fold cross-validation (*k* = 10) and resampling methods were used for model training, while the test set was used for parameter optimization. System performance was evaluated on both the test and validation sets. The clinical value of the prediction models was assessed based on three criteria: AUC, calibration, and clinical utility. AUC represents the area under the ROC curve. Calibration was evaluated using calibration curves and the Brier score to assess the agreement between predicted probabilities and actual outcomes and the overall prediction error. To evaluate clinical benefit, Decision Curve Analysis (DCA) was used to calculate the net benefit across different probability thresholds. Models were built and evaluated using Python 3.10 software.

### SHAP interpretability analysis

SHapley Additive exPlanations (SHAP) is a method for *post-hoc* interpretation of models based on Shapley values from game theory. It calculates the marginal contribution of each feature variable to the model's output, providing explanations at both global and local levels for “black-box models” (14). A higher SHAP value indicates a greater positive influence of that variable on the model output; conversely, a lower SHAP value indicates the opposite influence (15).

### Statistical methods

This study was conducted and reported in strict accordance with the Transparent Reporting of a multivariable prediction model for Individual Prognosis Or Diagnosis (TRIPOD) statement. Categorical variables are presented as frequencies (percentages) and compared using Chi-square test or Fisher's exact test as appropriate. Continuous variables are presented as mean (standard deviation) or median [interquartile range] based on normality and homogeneity of variance, and compared using *t*-test or Wilcoxon rank-sum test, respectively. A two-sided *P*-value < 0.05 was considered statistically significant. Statistical analyses were performed using R 4.5.1 (R Foundation for Statistical Computing) and Python 3.10 (Python Software Foundation).

## Results

### Patient demographics and clinical characteristics

As shown in Table 1, 2,494 immunosuppressed sepsis patients from the MIMIC-IV database were included in the study, of whom 833 (33.4%) died within 30 days. Compared to the survival group, the non-survival group was older (71.00 vs. 67.00 years, *P* < 0.05) and had higher SOFA scores (11.00 vs. 8.00, *P* < 0.05). Significant differences (*P* < 0.05) were observed between the survival and non-survival groups in Weight, APS_III, SAPS_II, GCS, Charlson comorbidity index, RBC, MCH, MCV, MCHC, Cr, BUN, PT, PLT, INR, AST, and Urine output. Significant differences (*P* < 0.05) were also found in the prevalence of comorbidities such as hypertension, heart disease, liver disease, kidney disease, and lung disease, as well as in the use of vasoactive drugs, invasive ventilation, continuous renal replacement therapy, and renal replacement therapy. No significant differences (*P* > 0.05) were found in sex distribution, diabetes prevalence, height, WBC, or ALT.

**Table 1:** Baseline demographic, clinical, and laboratory characteristics of immunocompromised sepsis patients in the derivation (MIMIC-IV).

Characteristics	Survival	Death	*P*
	(*n* = 1,661)	(*n* = 833)	
Demographics			
Male (%)	935 (56.29)	466 (55.94)	0.902
Age (years)	67.00 [56.00, 77.00]	71.00 [61.00, 81.00]	<0.001
height (cm)	170.00 [160.00, 178.00]	168.00 [160.00, 175.00]	0.073
weight (kilograms)	80.60 [67.00, 98.30]	77.20 [62.50, 95.00]	<0.001
Comorbidities			
Hypertension (%)	597 (35.94)	253 (30.37)	0.006
Diabetes (%)	600 (36.12)	288 (34.57)	0.473
Heart disease (%)	659 (39.67)	405 (48.62)	<0.001
Liver disease (%)	280 (16.86)	237 (28.45)	<0.001
Renal disease (%)	449 (27.03)	275 (33.01)	0.002
Pulmonary disease (%)	438 (26.37)	262 (31.45)	0.09
Clinical situation			
APS_III	56.00 [44.00, 70.00]	73.00 [58.00, 92.00]	<0.001
SAPS_II	43.00 [35.00, 52.00]	55.00 [46.00, 65.00]	<0.001
SOFA	8.00 [5.00, 11.00]	11.00 [8.00, 15.00]	<0.001
GCS	13.00 [9.00, 14.00]	10.00 [4.00, 14.00]	<0.001
CCI	5.00 [3.00, 7.00]	6.00 [5.00, 9.00]	<0.001
Lab test			
WBC (10^9^/L)	15.00 [9.50, 21.50]	15.20 [9.50, 22.70]	0.584
RBC (10^9^/L)	3.43 [2.97, 4.01]	3.33 [2.81, 3.91]	<0.001
HCT(%)	31.30 [27.20, 36.10]	31.40 [26.70, 36.00]	0.631
MCH (pg)	29.70 [27.90, 31.20]	30.10 [28.50, 32.00]	<0.001
MCV (fL)	92.00 [87.00, 96.00]	94.00 [89.00, 100.00]	<0.001
MCHC g/L)	32.27 (1.69)	31.95 (1.79)	<0.001
Cr (mg/dL)	1.30 [0.90, 2.20]	1.80 [1.10, 3.00]	<0.001
BUN (mg/dL)	27.00 [18.00, 47.00]	38.00 [24.00, 62.00]	<0.001
PT (s)	15.50 [13.50, 19.10]	17.10 [14.20, 24.00]	<0.001
PLT (10^9^/L)	222.00 [136.00, 338.00]	192.00 [105.00, 316.00]	<0.001
INR	1.40 [1.20, 1.80]	1.60 [1.30, 2.20]	<0.001
ALT (U/L)	38.00 [20.00, 109.00]	40.00 [20.00, 133.00]	0.152
AST (U/L)	56.00 [27.00, 133.00]	75.00 [35.00, 220.00]	<0.001
Urine (mL)	500.00 [300.00, 675.00]	270.00 [105.00, 500.00]	<0.001
Treatment			
Vasoactive_Agents (%)	1,172 (70.56)	650 (78.03)	<0.001
InvasiveVent (%)	985 (59.30)	638 (76.59)	<0.001
CRRT (%)	193 (11.62)	236 (28.33)	<0.001
RRT (%)	277 (16.68)	281 (33.73)	<0.001

Continuous variables are presented as mean ± standard deviation (SD) or median [interquartile range (IQR)]. Categorical variables are presented as counts (percentage). Comparisons between groups were performed using the Student's *t*-test or Mann–Whitney *U*-test for continuous variables, and the Chi-square test or Fisher's exact test for categorical variables, as appropriate. SOFA, Sequential Organ Failure Assessment; APS-III, Acute Physiology Score III; SAPS-II, Simplified Acute Physiology Score II; GCS, Glasgow Coma Scale; CCI, Charlson Comorbidity Index.

### Multi-model evaluation analysis

**Figure 4:**
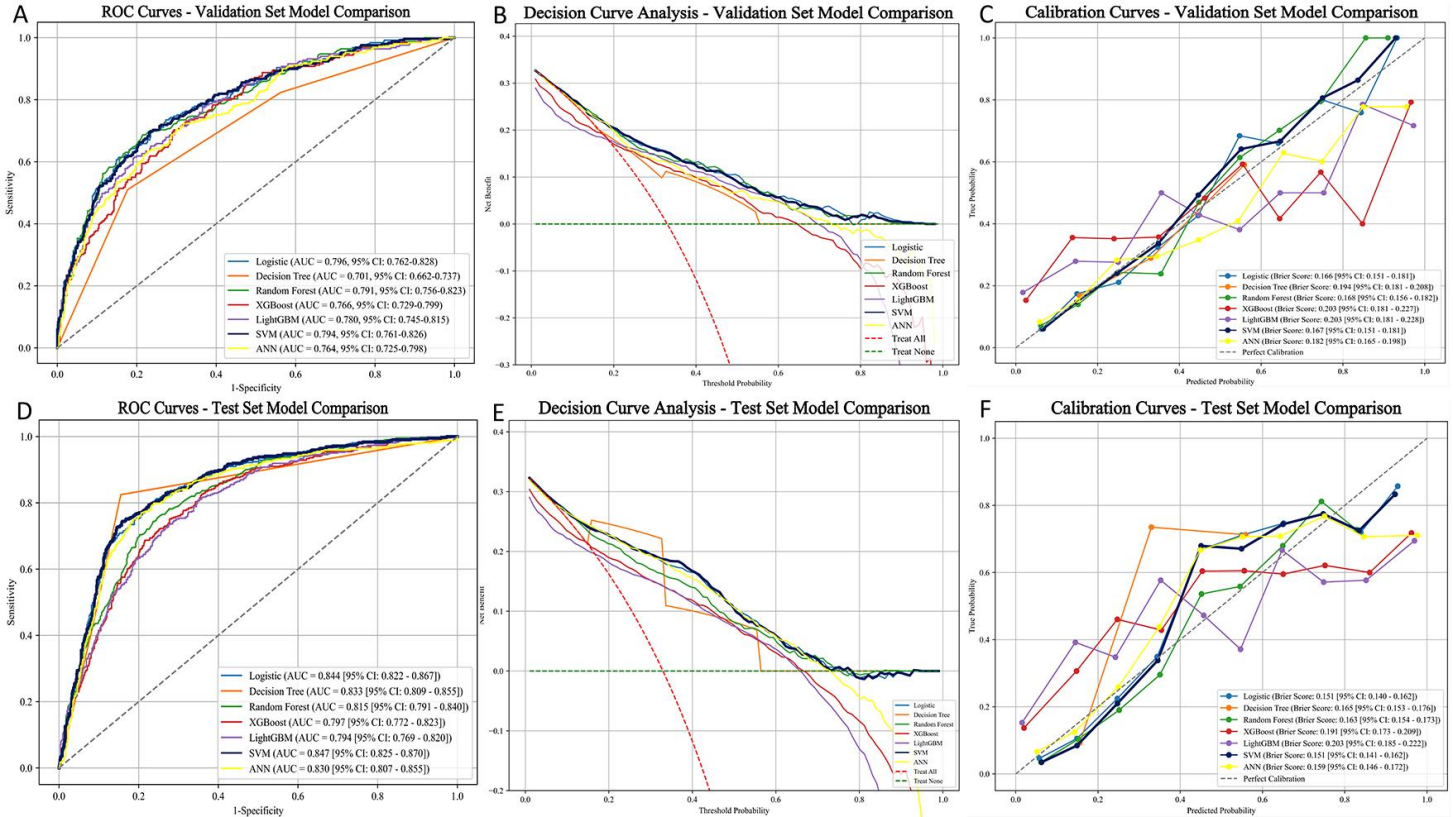
Comprehensive performance discrimination, clinical utility, and calibration profiles of the seven machine learning models. **(A)** ROC curves in the validation set; **(B)** decision curve analysis (DCA) in the validation set; **(C)** calibration curves in the validation set; **(D)** ROC curves in the multi-center external test set; **(E)** DCA in the test set; **(F)** calibration curves in the test set. The SVM model demonstrated superior and highly stable clinical net benefit and discriminative capacity across both cohorts. ROC, receiver operating characteristic; AUC, area under the ROC curve; DCA, decision curve analysis; SVM, support vector machine; ANN, artificial neural network; CI, confidence interval.

Seven machine learning models were built using clinical data from the first 6 h of ICU admission to predict 30-day mortality risk in immunosuppressed sepsis patients. As shown in Table 2 and Figure 4, in the validation set, Logistic Regression (LR) had an AUC of 0.796 (95% CI: 0.762–0.828), an F1 score of 0.635, and a Brier score of 0.166; SVM had an AUC of 0.794 (95% CI: 0.761–0.826), an F1 score of 0.631, and a Brier score of 0.167; performance metrics for other models are shown in the respective table and figure. Comparing all parameters, both LR and SVM models showed relatively high AUC and F1 scores, and low Brier scores in the validation set, and also demonstrated stable predictive performance in the test set (*n* = 1,280) (Figures 4A,D). Calibration curves for both models demonstrated favorable and consistent calibration performance across the predicted probability spectrum (Figures 4C,F), although minor fluctuations were noted in the high-risk strata, which are consistent with the limited sample density in these populations. Decision Curve Analysis indicated good clinical utility for both (Figures 4B,E). However, when the LR model was calibrated using the Bootstrap method (Supplementary Figure S3), its predictions were found to overestimate risk, leading to its exclusion. Therefore, the SVM model was selected as the optimal prediction model.

**Table 2:** Performance data of the seven machine learning models in the validation and test sets.

Dataset	Metrics	Logistic regression	Decision tree	Random forest	XGBoost	LightGBM	SVM	ANN
Validation set	AUC	0.796	0.701	0.791	0.766	0.780	0.794	0.764
	PR-AUC	0.682	0.536	0.681	0.637	0.629	0.678	0.610
	Sensitivity	0.755	0.823	0.759	0.578	0.614	0.743	0.743
	Specificity	0.689	0.438	0.631	0.787	0.807	0.695	0.616
	F1 score	0.635	0.559	0.608	0.577	0.614	0.631	0.592
	Brier score	0.166	0.194	0.168	0.203	0.203	0.167	0.182
Test set	AUC	0.844	0.833	0.815	0.797	0.794	0.847	0.830
	PR-AUC	0.692	0.606	0.653	0.614	0.603	0.692	0.619
	Sensitivity	0.791	0.825	0.839	0.626	0.630	0.806	0.763
	Specificity	0.770	0.845	0.627	0.808	0.805	0.754	0.796
	F1 score	0.701	0.771	0.646	0.620	0.622	0.699	0.701
	Brier score	0.151	0.165	0.163	0.191	0.203	0.151	0.159

### Model interpretation and application

**Figure 5:**
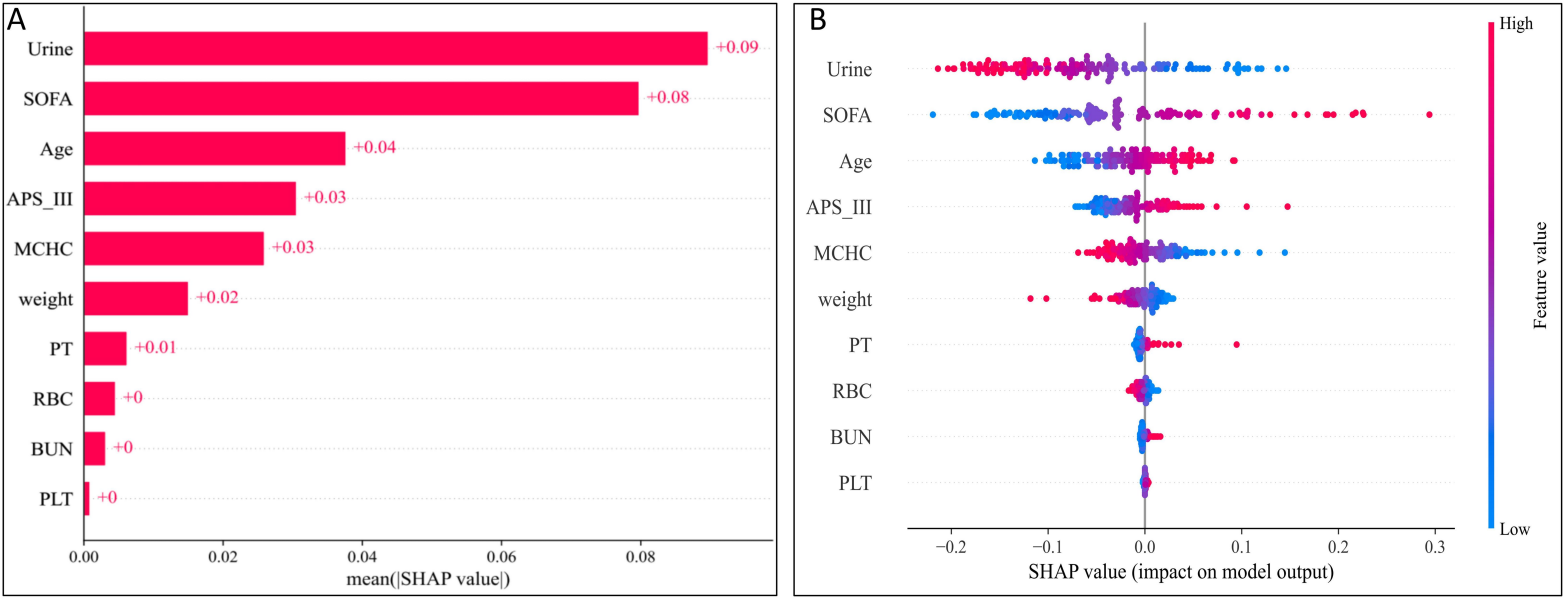
Summary plot of SHAP (SHapley Additive exPlanations) values for the top 10 clinical features in the final SVM model. Features are ranked by their overall impact on predicting 30-day mortality risk, with red indicating higher feature values and blue indicating lower values. **(A)** SHAP feature importance bar plot; **(B)** SHAP summary beeswarm plot.

The contribution of each variable to the prediction outcome was quantified using SHAP values. A higher SHAP value indicates a higher probability of the positive event (death). Figure 5A visualizes the importance of features using SHAP summary plots. The results show the top ten feature variables associated with 30-day mortality in immunosuppressed sepsis patients: Weight, APS-III score, Urine output, PT, BUN, SOFA score, RBC, PLT, Age, and MCHC. This ranking is further corroborated by the SHAP beeswarm plot (Figure 5B), which visually displays the impact of each feature on the model output. When a feature has a high value and a SHAP value >0, the 30-day mortality risk increases; when a feature has a high value and a SHAP value <0, the risk decreases. Taking SOFA score as an example, red dots to the right of the zero line represent higher SOFA values, which increase the 30-day mortality risk.

**Figure 6:**
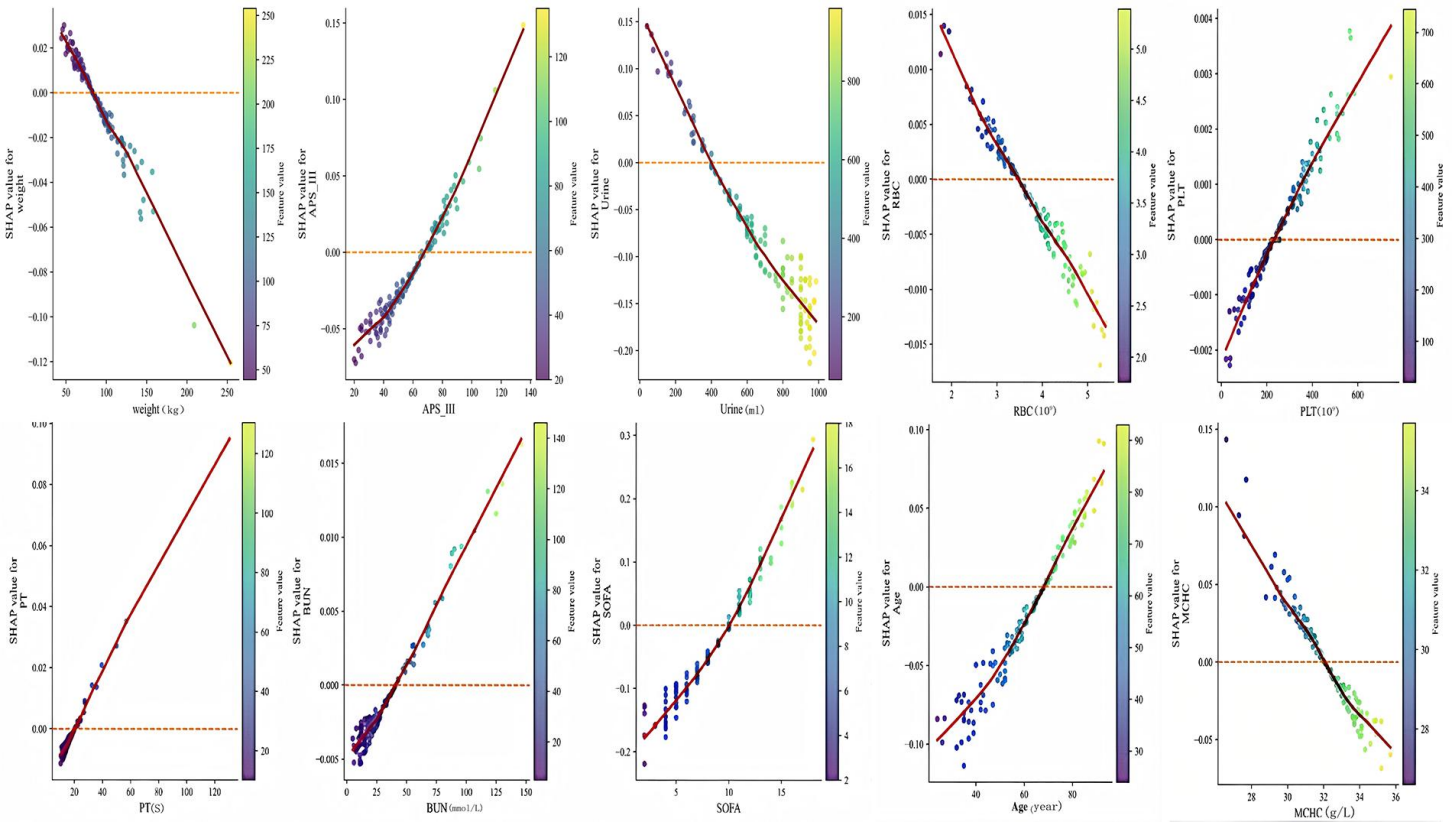
SHAP dependence plots for features in the SVM model: the *X*-axis shows the feature value, the *Y*-axis shows the corresponding SHAP value, reflecting the contribution of each feature to the model. The curve shows the trend of the relationship between feature value and SHAP value.

As shown in Figure 6, SHAP dependence plots were used to analyze the impact of features at the factor level on the prediction model. In the SVM model, all 10 variables showed a linear relationship with 30-day mortality risk. Weight, Urine output, RBC, and MCHC were negatively correlated with 30-day mortality risk, while APS_III, PT, BUN, SOFA score, PLT, and Age were positively correlated with 30-day mortality risk.

**Figure 7:**
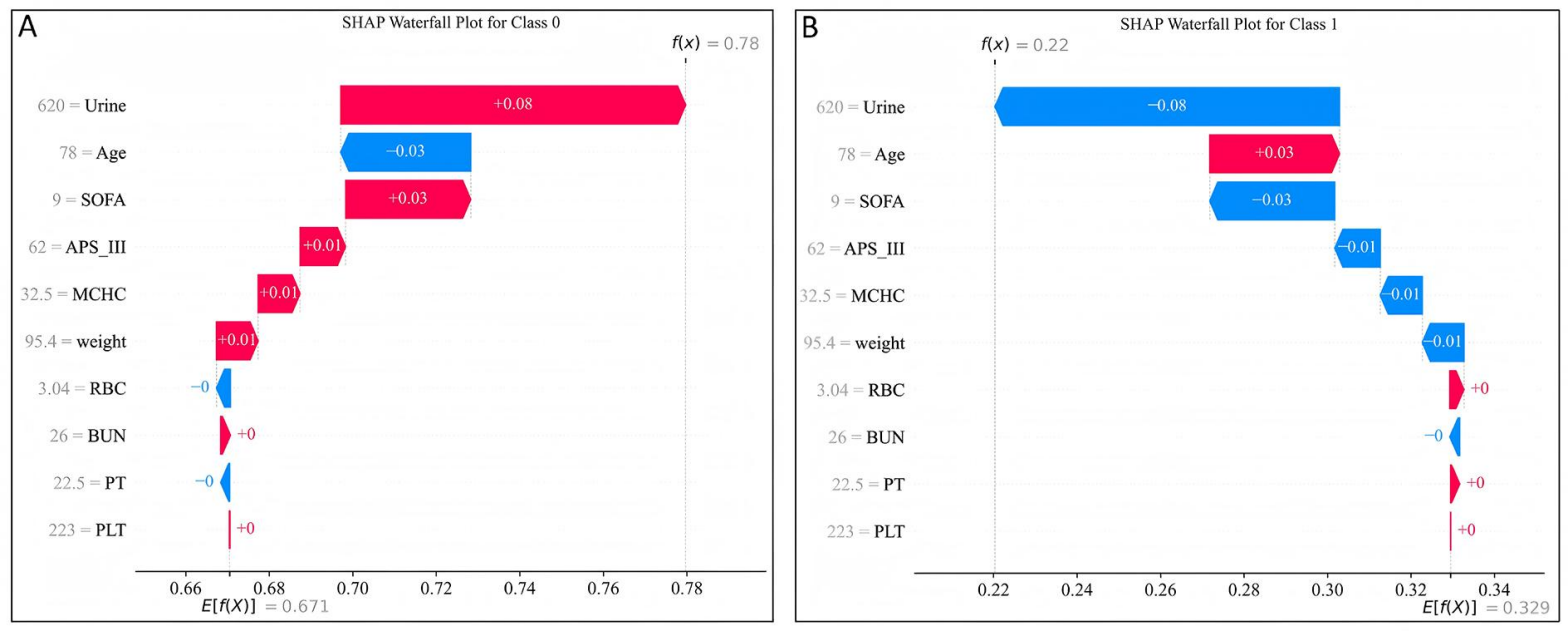
Waterfall plots. Red indicates a positive influence of the feature on the predicted 30-day mortality risk, blue indicates the opposite influence. (**A**) The final value for this individual is 0.780, higher than the base value (0.671), suggesting an increased probability of 30-day survival for this patient; (**B**) Shows the same individual, with a final value of 0.220, lower than the base value (0.329), suggesting a decreased 30-day mortality risk for this patient. **(A)** SHAP waterfall plot for Class 0; **(B)** SHAP waterfall plot for Class 1.

Furthermore, we analyzed a typical case using waterfall plots to demonstrate the model's interpretability at the individual level: Figure 7A shows the probability prediction for a negative event (30-day survival), and Figure 7B shows the probability prediction for a positive event (30-day death) for the same case. The plot shows the impact of key features on the prediction outcome for this individual. Compared to the base value (average prediction), factors pushing the prediction score higher are marked in red, while those lowering the score are in blue. The length of the arrow visually represents the magnitude of the impact—longer arrows indicate more significant influence.

## Discussion

Based on the MIMIC-IV and eICU databases, this study successfully developed and validated a machine learning model using clinical data from the first 6 h of ICU admission to predict 30-day mortality risk in immunosuppressed sepsis patients. Unlike previous studies focusing on the general sepsis population, we specifically targeted this special and high-risk immunosuppressed subgroup, aiming to address the more complex clinical challenge of prognosis determination in these patients. By systematically comparing multiple machine learning algorithms, we found that the Support Vector Machine (SVM) model demonstrated the best predictive performance (AUC = 0.796), outperforming commonly used models like Logistic Regression, Random Forest, and XGBoost. The final model incorporated 10 clinical and laboratory indicators readily available within 6 h of ICU admission, including Weight, APS-III score, Urine output, PT, BUN, SOFA score, RBC, PLT, Age, and MCHC, highlighting the model's potential for immediate bedside application. Compared to Zhong et al. (16), who utilized “worst values” during the ICU stay, our model strictly employs data from the first 6 h. This aligns with the “golden hour” principle, enabling early risk stratification upon admission before organ dysfunction peaks. Additionally, we validated our model using the multi-center eICU database, demonstrating superior generalizability over their single-center MIMIC-IV validation.

The 10 predictors selected in this study have clear clinical pathophysiological significance, collectively depicting key dimensions determining the prognosis of immunosuppressed sepsis patients. SOFA score and APS-III score are classic indicators for assessing the severity of organ dysfunction and acute physiological disturbance, and have been proven to be strong predictors of mortality in multiple sepsis prognosis models (17, 18). Six-hour urine output is a sensitive indicator for assessing renal perfusion and early acute kidney injury, and the kidney is one of the most vulnerable organs in sepsis; reduced urine output is closely associated with poor outcomes (19). Age is a fundamental factor reflecting physiological reserve and frailty, and elderly immunosuppressed patients have poorer tolerance to the insult of sepsis (20).

It is particularly noteworthy that our model highlights the central role of coagulation disorders in the prognosis of immunosuppressed sepsis patients. Prolonged PT suggest coagulopathy, which plays a core role in the progression of sepsis to multiple organ failure and is a key manifestation of sepsis-induced coagulopathy (SIC) (21, 22). A decreased platelet count reflects continuous consumption during microvascular thrombosis and microcirculatory impairment, a hallmark of severe inflammation and progressive multi-organ failure (23, 24). In our cohort, the death group presented with significantly lower baseline platelet counts compared to the survival group (*p* < 0.001), highlighting that accelerated platelet consumption directly closely correlates with an increased risk of mortality. Including this variable in the model enhances risk-stratification specificity, because platelet elevation is both more pronounced and more sustained in sepsis patients than in those with uncomplicated infection. BUN level not only reflects prerenal azotemia and possible dehydration but may also indirectly suggest potential complications like gastrointestinal bleeding, which carries a higher risk in patients receiving immunosuppressive therapy. The inclusion of RBC and MCHC suggests that anemia and abnormal red blood cell parameters may be associated with prognosis in immunosuppressed sepsis patients. Anemia is extremely common in critically ill patients, potentially related to inflammation-suppressed erythropoiesis, hemodilution, and blood loss; its severity affects tissue oxygen delivery and consequently outcomes (25, 26). While we observed statistically significant differences in RBC and MCHC levels between survivors and non-survivors, it is important to note that the absolute magnitudes of these differences are numerically small, and their clinical utility as independent diagnostic markers is likely limited. Nevertheless, within our multivariable machine learning framework, these parameters demonstrate incremental prognostic value. This suggests that these markers, rather than serving as primary diagnostic targets, function as vital components within our composite risk-prediction model by reflecting the patient's underlying physiological baseline and capacity to cope with septic insults. Interestingly, our model identified body weight as a highly important predictor of 30-day mortality. In the early hours of ICU admission, body weight carries profound pathophysiological implications beyond simple baseline biometrics. First, acute weight changes during the early phase often act as a proxy for fluid overload (27), which impairs tissue perfusion and worsens organ failure—a complication to which immunocompromised individuals are highly vulnerable. Second, lower baseline weight frequently underscores underlying nutritional depletion or frailty related to immunosuppressive states, signaling limited physiological resilience against infection (28). Lastly, body weight heavily dictates the pharmacokinetics of life-saving medications, where inappropriate dosing impacts survival. Consequently, body weight provides vital additive value alongside traditional severity scores for robust risk stratification (29).

From a methodological perspective, the superior performance of the SVM model may stem from its ability to handle complex linear and nonlinear relationships in high-dimensional feature spaces. The pathophysiology of immunosuppressed sepsis patients is extremely complex, with interactions between systems exhibiting nonlinear characteristics. SVM, using kernel tricks, can effectively capture this complexity, thereby providing higher discriminative accuracy in distinguishing between survivors and non-survivors (30, 31).

Placing our study in the context of existing literature, its innovation and value are mainly reflected in the following aspects. First, we specifically targeted the unique population of immunosuppressed sepsis patients. The immunosuppressed state significantly alters the host's response to infection, potentially reducing the performance of prediction models based on the general population (e.g., qSOFA, SOFA) in these patients (32). By incorporating risk factors specific to this population, our model could provides more precise individualized risk assessment. Second, we strictly limited the predictors to those available within 6 h of admission, ensuring the model's early warning value, aligning with the clinical need for intervention during the “golden hour” of sepsis, consistent with the early identification concept advocated by Seymour et al. (33). Su et al. used clinical data including vital signs, blood gas indicators, and treatment measures from the first 6 h of ICU admission to build a sepsis mortality prediction model (AUC: 0.74), but their model incorporated 20 variables, making clinical application cumbersome, and key indicators such as routine blood tests, biochemistry, and coagulation were not fully included, thus failing to fully reflect the true condition of sepsis patients. Furthermore, that model lacked external validation and visual interpretation (34), leaving its clinical predictive efficacy and generalizability requiring further confirmation. Our 10-variable model targets this high-mortality-risk group of immunosuppressed sepsis patients and, while maintaining clinical feasibility, potentially achieves higher mortality risk discrimination by including deeper pathophysiological information such as coagulation function.

Our model was evaluated on an external test set (eICU database), where its AUC increased to 0.847. Calibration curves and DCA both indicated good predictive performance, stability, and strong generalizability. This model could be integrated into hospital electronic medical record systems to automatically calculate the 30-day mortality risk for immunosuppressed sepsis patients after admission. High-risk patients could be flagged, triggering closer monitoring, earlier organ function support, more aggressive anti-infective therapy, or multidisciplinary consultations. In particular, the highlighted abnormal coagulation indicators in the model might suggest the potential benefit of interventions targeting coagulation disorders (e.g., anticoagulant therapy) for these patients (35), although this requires confirmation through prospective studies.

This study has several limitations. First, we did not include some potentially important variables, such as specific types of immunosuppressive drugs, microbiological results, or dynamic inflammatory markers (e.g., procalcitonin). Second, the populations in the eICU and MIMIC databases are predominantly from North America, and the model's promotion and application need validation in other populations. Third, although the MIMIC-IV and eICU databases encompass diverse patient populations, we did not formally evaluate model fairness or potential predictive heterogeneity across specific sociodemographic subgroups (e.g., race, ethnicity, or socioeconomic status). Future research directions should include: (1) Conducting prospective studies to evaluate its impact on real-world clinical decision-making; (2) Exploring the incorporation of dynamic data (e.g., trends in indicators) into the model to further enhance predictive performance; (3) validating the model's performance across different demographic populations to ensure equitable clinical utility and avoid algorithmic bias.

## Conclusion

In summary, using routine clinical data from the first 6 h of ICU admission, we developed a prediction model based on the SVM algorithm that accurately predicts 30-day mortality risk in immunosuppressed sepsis patients. The indicators included in the model are easily obtainable and emphasize the importance of organ function, disease severity, and particularly coagulation function in prognosis determination. This tool provides a promising approach for early risk stratification and individualized management of this high-risk population of immunosuppressed sepsis patients.

## Data Availability

The raw data supporting the conclusions of this article will be made available by the authors, without undue reservation.

## References

[1] Singer M Deutschman CS Seymour CW Shankar-Hari M Annane D Bauer M. The third international consensus definitions for sepsis and septic shock (sepsis-3). JAMA. (2016) 315(8):801–10. 10.1001/jama.2016.0287 26903338 PMC4968574

[2] Gao X Cai S Li X Wu G. Sepsis-induced immunosuppression: mechanisms, biomarkers and immunotherapy. Front Immunol. (2025) 16:1577105. 10.3389/fimmu.2025.1577105 40364841 PMC12069044

[3] Venet F Monneret G. Advances in the understanding and treatment of sepsis-induced immunosuppression. Nat Rev Nephrol. (2018) 14(2):121–37. 10.1038/nrneph.2017.165 29225343

[4] Lin H Yang L Fang J Gao Y Zhu H Zhang S. Clinical characteristics of bloodstream infection in immunosuppressed patients: a 5-year retrospective cohort study. Front Cell Infect Microbiol. (2022) 12:796656. 10.3389/fcimb.2022.796656 35444962 PMC9014008

[5] Mosevoll KA Hansen BA Gundersen IM Reikvam H Bruserud Ø Bruserud Ø. Systemic metabolomic profiles in adult patients with bacterial sepsis: characterization of patient heterogeneity at the time of diagnosis. Biomolecules. (2023) 13(2):223. 10.3390/biom13020223 36830594 PMC9953377

[6] Shillan D Sterne JAC Champneys A Gibbison B. Use of machine learning to analyse routinely collected intensive care unit data: a systematic review. Crit Care. (2019) 23(1):284. 10.1186/s13054-019-2564-9 31439010 PMC6704673

[7] Yan MY Gustad LT Nytrø Ø. Sepsis prediction, early detection, and identification using clinical text for machine learning: a systematic review. J Am Med Inform Assoc. (2022) 29(3):559–75. 10.1093/jamia/ocab236 34897469 PMC8800516

[8] Johnson AEW Bulgarelli L Shen L Gayles A Shammout A Horng S. MIMIC-IV, a freely accessible electronic health record dataset. Sci Data. (2023) 10(1):1. 10.1038/s41597-022-01899-x 36596836 PMC9810617

[9] Pollard TJ Johnson AEW Raffa JD Celi LA Mark RG Badawi O. The eICU Collaborative Research Database, a freely available multi-center database for critical care research. Sci Data. (2018) 5:180178. 10.1038/sdata.2018.178 30204154 PMC6132188

[10] Freifeld AG Bow EJ Sepkowitz KA Boeckh MJ Ito JI Mullen CA. Clinical practice guideline for the use of antimicrobial agents in neutropenic patients with cancer: 2010 update by the Infectious Diseases Society of America. Clin Infect Dis. (2011) 52(4):e56–93. 10.1093/cid/cir073 21258094

[11] Dropulic LK Lederman HM. Overview of infections in the immunocompromised host. Microbiol Spectr. (2016) 4(4). 10.1111/jcmm.12812 27726779 PMC8428766

[12] Xu Y Goodacre R. On splitting training and validation set: a comparative study of cross-validation, bootstrap and systematic sampling for estimating the generalization performance of supervised learning. J Anal Test. (2018) 2(3):249–62. 10.1007/s41664-018-0068-2 30842888 PMC6373628

[13] Han J Gondro C Reid K Steibel JP. Heuristic hyperparameter optimization of deep learning models for genomic prediction. G3 (Bethesda). (2021) 11(7):jkab032. 10.1093/g3journal/jkab032 33993261 PMC8495939

[14] Crombé A Kataoka M. Breast cancer molecular subtype prediction: improving interpretability of complex machine-learning models based on multiparametric-MRI features using SHapley Additive exPlanations (SHAP) methodology. Diagn Interv Imaging. (2024) 105(5):161–2. 10.1016/j.diii.2024.01.008 38365542

[15] Gadkari S De Oliveira RS Bolognesi S Puig S Nascimento EGS. Benchmarking machine learning algorithms for microbial electromethanogenesis: a comprehensive assessment with SHapley Additive exPlanation-based insights. ACS Sustain Chem Eng. (2026) 14(1):363–75. 10.1021/acssuschemeng.5c09770 41541636 PMC12801386

[16] Zhong Z He H Lin Z. A retrospective cohort study on 28-day mortality in immunosuppressed sepsis: an interpretability-based predictive model using MIMIC-IV V2.2. Shock. (2026) 65(3):348–59. 10.1097/SHK.0000000000002721 40997317

[17] Marshall JC Deutschman CS. The multiple organ dysfunction syndrome: syndrome, metaphor, and unsolved clinical challenge. Crit Care Med. (2021) 49(9):1402–13. 10.1097/CCM.0000000000005139 34259449

[18] Lei M Liu X Cheng L Li Y Tang N Song J. An ensemble machine learning-based risk stratification tool for 30-day mortality prediction in critically ill cardiovascular patients. Cardiovasc Diabetol. (2025) 24(1):373. 10.1186/s12933-025-02911-5 41029382 PMC12487267

[19] Oami T Imaeda T Nakada T Abe T Takahashi N Yamao Y. Mortality analysis among sepsis patients in and out of intensive care units using the Japanese nationwide medical claims database: a study by the Japan Sepsis Alliance study group. J Intensive Care. (2023) 11(1):2. 10.1186/s40560-023-00650-x 36611188 PMC9826578

[20] Han Y Xie X Qiu J Tang Y Song Z Li W. Early prediction of sepsis associated encephalopathy in elderly ICU patients using machine learning models: a retrospective study based on the MIMIC-IV database. Front Cell Infect Microbiol. (2025) 15:1545979. 10.3389/fcimb.2025.1545979 40313459 PMC12043699

[21] Iba T Helms J Connors JM Levy JH. The pathophysiology, diagnosis, and management of sepsis-associated disseminated intravascular coagulation. J Intensive Care. (2023) 11(1):24. 10.1186/s40560-023-00672-5 37221630 PMC10202753

[22] Wada H Shiraki K Shimaoka M. The prothrombin time ratio is not a more effective marker for evaluating sepsis-induced coagulopathy than fibrin-related markers. J Thromb Haemost. (2020) 18(6):1506–7. 10.1111/jth.14766 32496020

[23] Chen J Gao X Shen S Xu J Sun Z Lin R. Association of longitudinal platelet count trajectory with ICU mortality: a multi-cohort study. Front Immunol. (2022) 13:936662. 10.3389/fimmu.2022.936662 36059447 PMC9437551

[24] Wen S Zhou S Wang W Qiu X Feng Y. Associations between hematologic parameters and all-cause death in individuals with cardio-renal-metabolic multimorbidity: a national cohort study. J Am Heart Assoc. (2025) 14(18):e041978. 10.1161/JAHA.125.041978 40908505 PMC12554417

[25] Mantovani A Busti F Borella N Scoccia E Pecoraro B Sani E. Elevated plasma hepcidin concentrations are associated with an increased risk of mortality and nonfatal cardiovascular events in patients with type 2 diabetes: a prospective study. Cardiovasc Diabetol. (2024) 23(1):305. 10.1186/s12933-024-02377-x 39154180 PMC11330614

[26] Raasveld SJ De Bruin S ReulandRaasveld SJ De Bruin S Reuland MC Van Den Oord C. Red blood cell transfusion in the intensive care unit. Jama. (2023) 330(19):1852–61. 10.1001/jama.2023.20737 37824112 PMC10570913

[27] Park I Lee JH Jang D-H Kim J Hwang BR Kim S. Assessment of body water distribution in patients with sepsis during fluid resuscitation using multi-frequency direct segmental bioelectrical impedance analysis. Clin Nutr. (2020) 39(6):1826–31. 10.1016/j.clnu.2019.07.022 31416662

[28] Saini A Nasser A-S Stewart CE. Waste management—cytokines, growth factors and cachexia. Cytokine Growth Factor Rev. (2006) 17(6):475–86. 10.1016/j.cytogfr.2006.09.006 17118696

[29] Jingyi S Cunliang G Biao C Yingguang X Jinluan M Xiaohua C. Vascular reactivity index as an effective predictor of mortality in patients with septic shock: a retrospective study. J Intensive Care Med. (2024) 39(8):794–800. 10.1177/08850666241233183 38465637

[30] Zheng Y Wang J Ling Z Zhang J Zeng Y Wang K. A diagnostic model for sepsis-induced acute lung injury using a consensus machine learning approach and its therapeutic implications. J Transl Med. (2023) 21(1):620. 10.1186/s12967-023-04499-4 37700323 PMC10498641

[31] Wang D Li J Sun Y Ding X Zhang X Liu S. A machine learning model for accurate prediction of sepsis in ICU patients. Front Public Health. (2021) 9:754348. 10.3389/fpubh.2021.754348 34722452 PMC8553999

[32] Gustot T. Multiple organ failure in sepsis: prognosis and role of systemic inflammatory response. Curr Opin Crit Care. (2011) 17(2):153–9. 10.1097/MCC.0b013e328344b446 21346564

[33] Seymour CW Gesten F Prescott HC Friedrich ME Iwashyna TJ Phillips GS. Time to treatment and mortality during mandated emergency care for sepsis. N Engl J Med. (2017) 376(23):2235–44. 10.1056/NEJMoa1703058 28528569 PMC5538258

[34] Su L Xu Z Chang F Ma Y Liu S Jiang H. Early prediction of mortality, severity, and length of stay in the intensive care unit of sepsis patients based on sepsis 3.0 by machine learning models. Front Med (Lausanne). (2021) 8:664966. 10.3389/fmed.2021.664966 34291058 PMC8288021

[35] Iba T Levi M Thachil J Helms J Scarlatescu E Levy JH. Communication from the Scientific and Standardization Committee of the International Society on thrombosis and haemostasis on sepsis-induced coagulopathy in the management of sepsis. J Thromb Haemost. (2023) 21(1):145–53. 10.1016/j.jtha.2022.10.022 36695377

