# Correction: Association between clinical characteristics within 6 h of ICU admission and 30-day mortality risk in immunocompromised sepsis patients: development and validation of a machine learning model based on the MIMIC-IV database

**DOI:** 10.3389/fdgth.2026.1936568

**Published:** 2026-07-31

**Authors:** Zhipeng Cheng, Xiuqing Ma, Weiying Duan, Zeyu Mou, Zhixin Liang

**Affiliations:** Department of Respiratory and Critical Care Medicine, First Medical Center, Chinese PLA General Hospital, Beijing, China

**Keywords:** eICU-CRD, immunosuppression, MIMIC-IV, mortality risk, prediction model, sepsis, support vector machine

There was a mistake in Figure 5 as published. Due to an upload error during the production stage, the feature labels and ordering in Figure 5B were incorrect. The corrected Figure 5 appears below.

**Figure 5:**
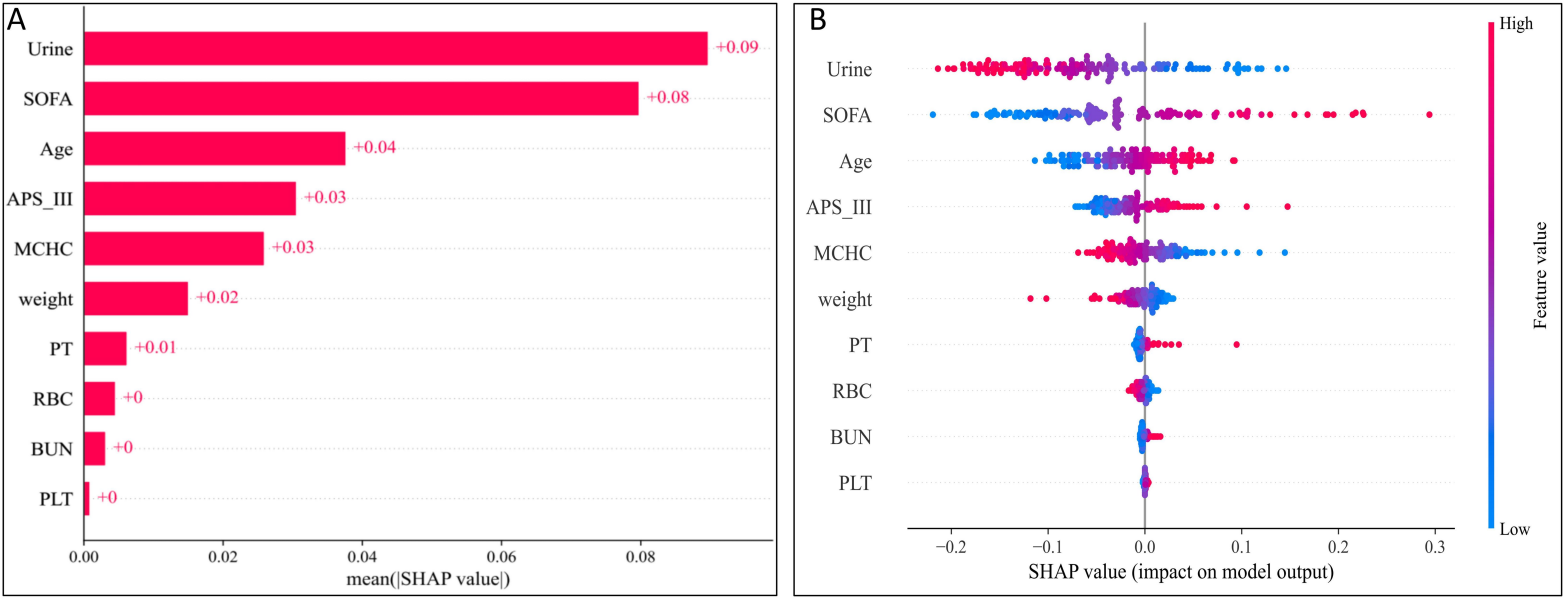


In the published article, there was a mistake in the text on page 5, where “PTT” was erroneously written instead of “PLT”.

A correction has been made to the Results section, paragraph 1:

“Weight, APS_III, SAPS_II, GCS, Charlson comorbidity index, RBC, MCH, MCV, MCHC, Cr, BUN, PT, PLT, INR, AST, and Urine output. Significant differences (*P* **<**0.05) were also found in the prevalence of comorbidities such as hypertension, heart disease, liver disease, kidney disease, and lung disease, as well as in the use of vasoactive drugs, invasive ventilation, continuous renal replacement therapy, and renal replacement therapy”.

The original version of this article has been updated.

