# Supplementary material for: Association between clinical characteristics within 6 h of ICU admission and 30-day mortality risk in immunocompromised sepsis patients: development and validation of a machine learning model based on the MIMIC-IV database
Source: Front Digit Health. 2026 Jul 13;8:1881138. doi: 10.3389/fdgth.2026.1881138 (PMC13402528; doi:10.3389/fdgth.2026.1881138)
Supplement: Supplementary file 1 [file Datasheet1.pdf]

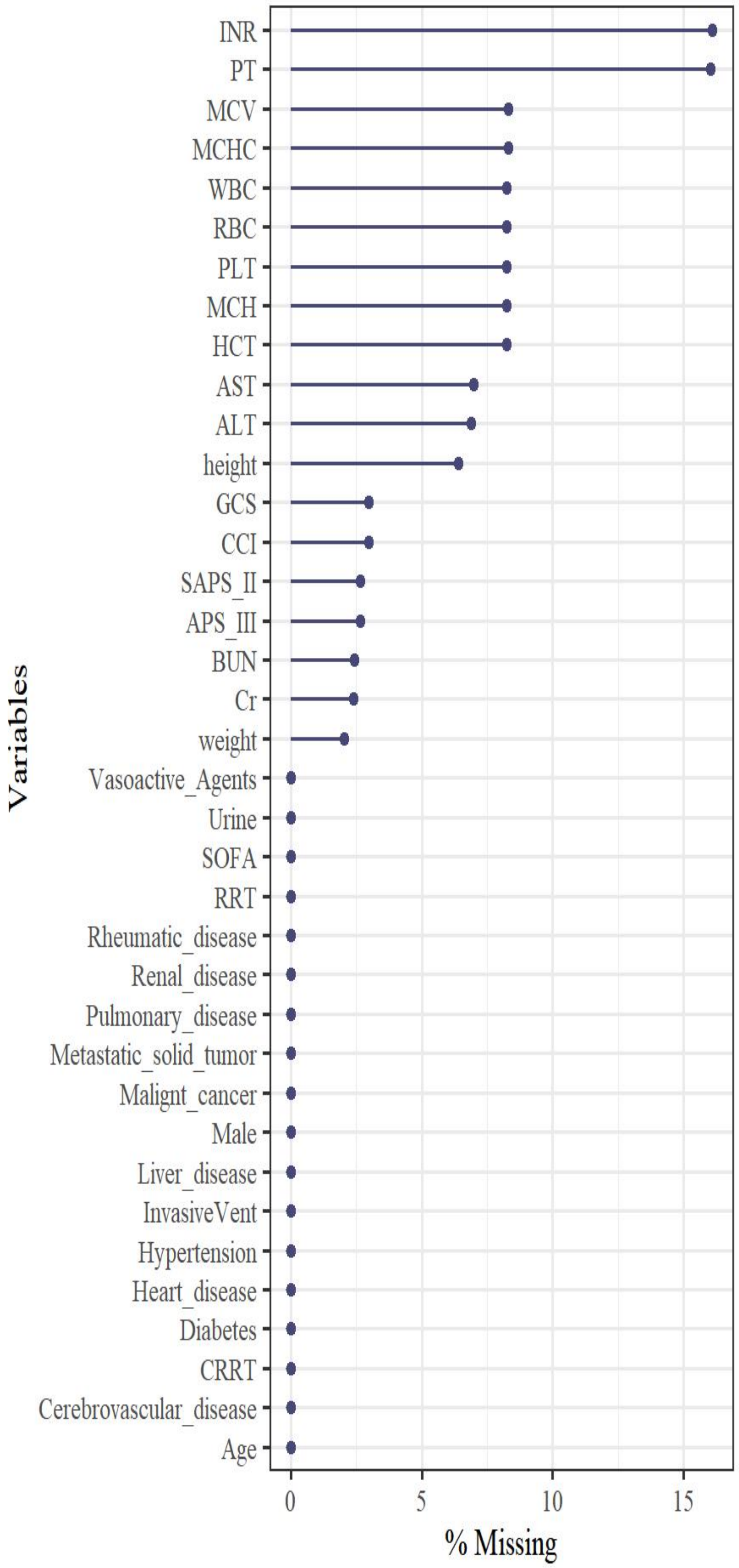

S1 Missing Data Proportion Plot

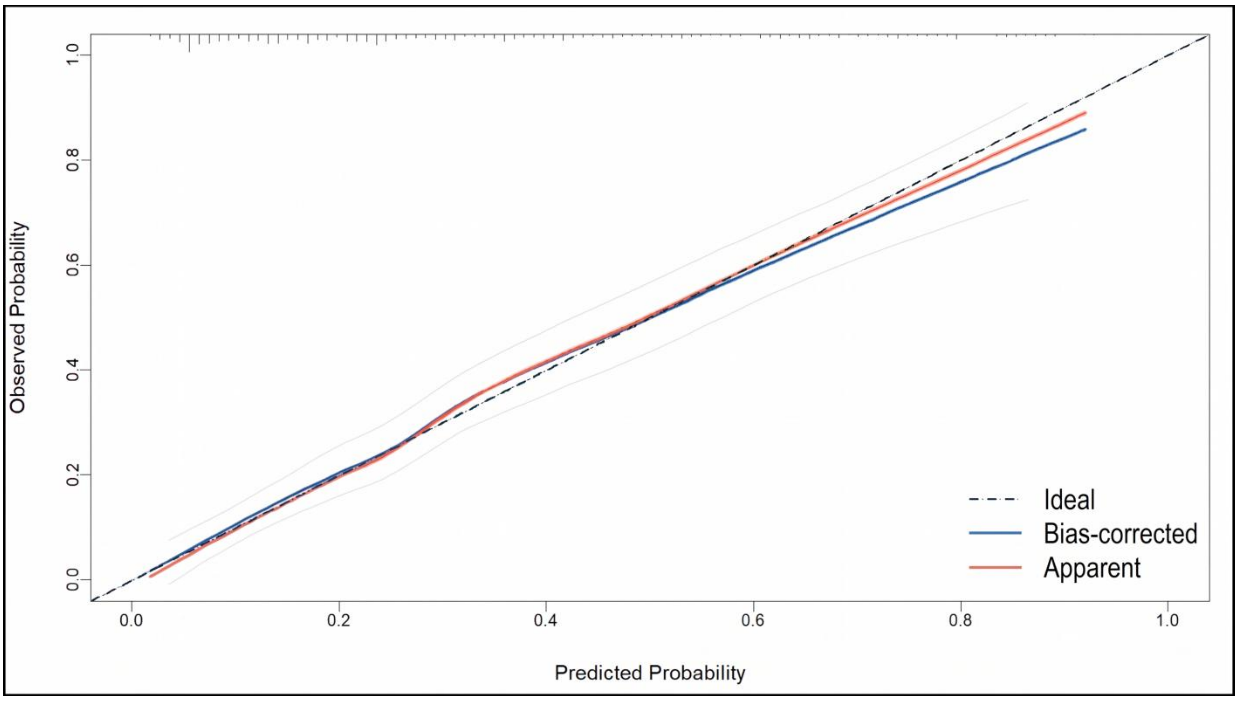

S2. the LR model calibration curves—including both the apparent curve (red) and the bias-corrected curve (blue)—deviate from the ideal line (dashed). Notably, the bias-corrected curve exhibits a more pronounced deviation, indicating a distinct risk of overestimation
